# Rapid Intraspecies Evolution of Fitness Effects of Yeast Genes

**DOI:** 10.1093/gbe/evac061

**Published:** 2022-04-28

**Authors:** Yayu Wang, Bei Jiang, Yue Wu, Xionglei He, Li Liu

**Affiliations:** MOE Key Laboratory of Gene Function and Regulation, State Key Laboratory of Biocontrol, School of Life Sciences, Sun Yat-sen University, Guangzhou 510275, China; MOE Key Laboratory of Gene Function and Regulation, State Key Laboratory of Biocontrol, School of Life Sciences, Sun Yat-sen University, Guangzhou 510275, China; MOE Key Laboratory of Gene Function and Regulation, State Key Laboratory of Biocontrol, School of Life Sciences, Sun Yat-sen University, Guangzhou 510275, China; MOE Key Laboratory of Gene Function and Regulation, State Key Laboratory of Biocontrol, School of Life Sciences, Sun Yat-sen University, Guangzhou 510275, China; MOE Key Laboratory of Gene Function and Regulation, State Key Laboratory of Biocontrol, School of Life Sciences, Sun Yat-sen University, Guangzhou 510275, China

**Keywords:** fitness effect, intraspecies variation, mutant phenotype, genetic background

## Abstract

Organisms within species have numerous genetic and phenotypic variations. Growing evidences show intraspecies variation of mutant phenotypes may be more complicated than expected. Current studies on intraspecies variations of mutant phenotypes are limited to just a few strains. This study investigated the intraspecies variation of fitness effects of 5,630 gene mutants in ten *Saccharomyces cerevisiae* strains using CRISPR–Cas9 screening. We found that the variability of fitness effects induced by gene disruptions is very large across different strains. Over 75% of genes affected cell fitness in a strain-specific manner to varying degrees. The strain specificity of the fitness effect of a gene is related to its evolutionary and functional properties. Subsequent analysis revealed that younger genes, especially those newly acquired in *S. cerevisiae* species, are more likely to be strongly strain-specific. Intriguingly, there seems to exist a ceiling of fitness effect size for strong strain-specific genes, and among them, the newly acquired genes are still evolving and have yet to reach this ceiling. Additionally, for a large proportion of protein complexes, the strain specificity profile is inconsistent among genes encoding the same complex. Taken together, these results offer a genome-wide map of intraspecies variation for fitness effect as a mutant phenotype and provide an updated insight on intraspecies phenotypic evolution.

**Significance**

It is usually believed that the genotype–phenotype relationship is largely conserved within species. Is this really true? Compared with intraspecies genetic evolution, intraspecies phenotypic evolution has not yet been systematically explored. Current studies are limited to several single genes or just a very few strains. To address this, we estimated the fitness effects of 5,630 gene mutants in ten *Saccharomyces cerevisiae* strains using CRISPR–Cas9 screening. We showed that the variability of fitness effects of considerable genes is very large across different strains, which support that phenotypic evolution may obey different laws of genotypic evolution.

## Introduction

A fundamental assumption of evolutionary biology is that orthologs, which are homologs derived from a single ancestral gene, typically retain similar functions (Koonin and Galperin 2003; Siepel et al. 2005). But at the same time, species-specific phenotypes such as essentiality, response to stress conditions, and drug resistance, have been observed even in closely related species of yeast and bacteria (Apjok et al. 2019; Sanchez et al. 2019; Doughty et al. 2020). This interspecies variability of functions for the same genes is not beyond expectations and reflects the adaptive evolution of different species (Pfennig et al. 2010; Zamudio et al. 2016). In fact, the variability is also existed within species. Different phenotypes have been reported for the same gene mutant within a species in mice, *Drosophila melanogaster*, and yeast (Threadgill et al. 1995; Dworkin et al. 2009; Liu et al. 2020). Death which is brought by epidermal growth factor receptor (*EGFR*) deficiency can occur at peri-implantation, midgestation, or 3 weeks after birth in three mouse strains (Threadgill et al. 1995). The *scalloped^E3^* mutation has distinct effects on wing morphology in two strains of *D. melanogaster* (Dworkin et al. 2009). In a previous study of ours, the deletion effects of *HAP4* were examined in three *Saccharomyces cerevisiae* (*S. cerevisiae*) strains, and a fairly large part of the deletion effects was found to be poorly conserved (Liu et al. 2020).

These are not exceptional cases, and intraspecies variations of mutant phenotypes exist even on a genome-wide scale. For two *S. cerevisiae* strains with ∼99.7% sequence similarity, comparative gene deletion experiments showed that 894 genes were essential in both strains, but that 44 genes were essential in only one strain and 13 genes were essential in only the other strain (Dowell et al. 2010). Galardini et al. (2019) measured growth in 38 conditions for 3,786 gene knockouts in four *S. cerevisiae* strains, and found only 9–24% of gene deletion phenotypes significantly conserved in all four backgrounds. The effect of genetic background on loss-of-function phenotypes was investigated in two isolates of *Caenorhabditis elegans* as well, and ∼20% genes were found to have different severity of phenotypes in two isolates (Vu et al. 2015). All these studies suggest that intraspecies variations of genotype-to-phenotype relationships may be more complicated than expected, and remind us to consider the impact of genetic backgrounds to mutant phenotypes. In fact, organisms within species have numerous genetic variations (The International HapMap Consortium 2005; Clark et al. 2007; Frazer et al. 2007; Schacherer et al. 2009). Intraspecies phenotypic variations are produced by these genetic variations together with environmental factors (Nei 2007), and have been observed in genetically diverse yeast strains, *Arabidopsis thaliana* inbred lines, and humans (Sabatti et al. 2009; Atwell et al. 2010; Skelly et al. 2013).

**Fig. 1.**
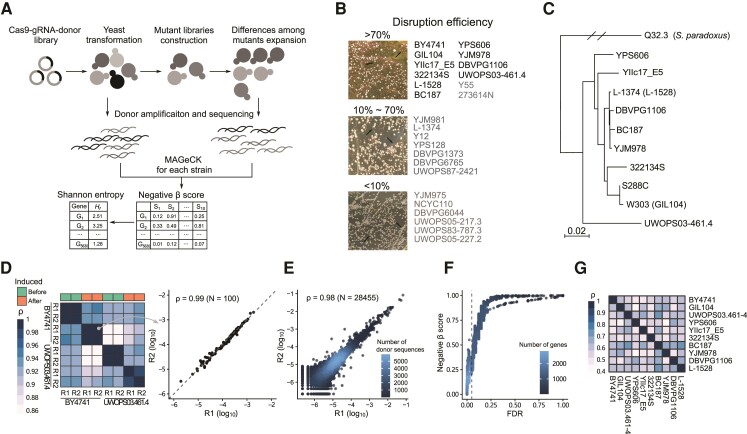
Genome-wide CRISPR–Cas9 screening in various *Saccharomyces cerevisiae* strains. (*A*) CRISPR–Cas9 screening workflow. The sgRNAs with donor sequences targeting different genes were constructed into the vectors with a Cas9 expression cassette. The pooled library was delivered into each yeast strain. The expression of Cas9 in each yeast library was induced to generate mutants. The abundance differences among mutants were enlarged by expanding culturing. Here, we used the donor sequence as a unique barcode for each gene. Donor sequences are longer than guide sequences, which provide higher accuracy in mapping. The cells of various strains in different stages were collected and used to extract plasmids. Donor sequences were amplified and sequenced. Reads were mapped and analyzed by MAGeCK to quantify the variations of abundances of donor sequences for each strain. The negative *β*-score of each gene was taken as the proxy of fitness effect after disruption. Then, the value of Shannon entropy (*H_f_*) of each gene was calculated, which indicated the strain specificity of fitness effect for each gene. (*B*) Disruption efficiency of CRISPR–Cas9 system targeting *ADE2* in 25 *S. cerevisiae* strains. Loss-of-function mutations in *ADE2* can lead to the accumulation of red pigment in cells because *ADE2* is required for adenine biosynthesis. The red clones (black arrows) represented the successful editing events. Strains labeled black were chosen for the next experiments. (*C*) Phylogenetic tree of ten *S. cerevisiae* strains with a *S. paradoxes* strain Q32.3 as an outgroup. (*D*) Spearman’s correlation coefficients of relative abundances of 100 random donor sequences among replicates before and after induced incubation. The relative abundance was estimated by the proportion of donor sequence counts in the total counts in each sample. The heatmap (left) shows Spearman’s correlation coefficients derived from BY4741 and UWOPS03-461.4, and the scatter plot (right) is the relative abundance of two replicates after induced incubation in BY4741. (*E*) The relative abundance of 28,455 donor sequences of two replicates after induced incubation in BY4741. The gradient represents the density of donor sequences. (*F*) The relationship between negative *β*-score and FDR in BY4741 derived from MAGeCK. The dashed line represents FDR = 0.05. The gradual blues represent the density of genes. (*G*) Heatmap of Spearman’s correlation coefficients of negative *β*-scores among 10 strains.

Current studies on intraspecies variations of mutant phenotypes are limited to just a few strains. In this study, we estimated the fitness effects of disruptions of ∼5,600 orthologous genes in ten *S. cerevisiae* strains by CRISPR–Cas9 screening. As a high-throughput genetic perturbation technology, CRISPR–Cas9 screening is widely used to identify genes essential for cell viability, host factors essential for influenza virus replication, human pluripotency-specific genes, and other functional elements (Shalem et al. 2014; Zhu et al. 2016; Haapaniemi et al. 2018; Han et al. 2018; Ihry et al. 2019). Here, we used a pooled single-guide RNA (sgRNA) library targeting ∼5,600 genes to generate large-scale loss-of-function mutants in various strains (fig. 1*A*). The strategy of a gRNA combined with a donor sequence was adopted to increase successful editing events (Bao et al. 2015; Guo et al. 2018) (see Materials and Methods). The donor sequence served as a barcode for each mutant, and the variation in barcode abundances before and after the generation of mutant reflected the fitness effect of gene disruption.

## Results

### Genome-Wide Measurement of Fitness Effects of Mutants in *S. cerevisiae* Strains

We first tested the disruption efficiency of the CRISPR–Cas9 system targeting the endogenous gene *ADE2* in 25 *S. cerevisiae* strains (see Materials and Methods, fig. 1*B*). The disruption efficiency of the system was different in the different strains, and ten strains with over 70% efficiency were selected (fig. 1*B*, supplementary file S1: fig. S1, Supplementary Material online). These strains have high genome similarity but different geographic origins or usages (Liti et al. 2009) (fig. 1*C*). The SNP densities of these strains are 0.5–2.7 per kilobase when compared with the reference strain S288C (the most widely studied strain of *S. cerevisiae*) (supplementary file S2, Supplementary Material online) (Bergström et al. 2014). To remove the effect of sequence divergence, CRISPR library was designed to target the conserved regions. In total 28,457 sgRNAs with donor sequences targeting 5,706 genes (∼86% of homologous genes in *S. cerevisiae* strains, ∼5–7 sequences per gene) were designed by considering their efficacy, specificity, positions in open reading frames (ORFs) (see Materials and Methods, supplementary file S3, Supplementary Material online). We also designed 100 random sgRNAs, each with 100 bp random donor sequences as a control. The sgRNA cassette, donor sequence, and Cas9 cassette were assembled into the background vector (see Materials and Methods).

The pooled library was delivered into each yeast strain, and each strain library was collected and incubated (fig. 1*A*). We optimized the induced incubation, including massive small-volume culturing to enhance the editing probability for each gene, and expanding culturing to enlarge the differences among mutants. The abundance of donor sequences before and after induced incubation was quantified by next-generation sequencing (NGS). There were two biological replicates for each strain in both conditions. As expected, the distributions of proportions of read counts for 100 random donor sequences in each replicate were quite consistent (fig. 1*D*, supplementary file S1: fig. S2, Supplementary Material online). Because the random donor sequences were nontarget, the distributions of abundances of them were stable in different strains (fig. 1*D*, supplementary file S1: fig. S2, Supplementary Material online). The editing events were highly robust in each strain as well. The correlations of proportions of read counts for donor sequences between two replicates after induced incubation were 0.92–0.96 (fig. 1*E*, supplementary file S1: fig. S3, Supplementary Material online). These results suggested that the assays were performed with high reproducibility.

Then the effects of gene disruptions created by the CRISPR–Cas9 system in each strain were analyzed by the model-based analysis of the genome-wide CRISPR–Cas9 knockout (MAGeCK) program. MAGeCK assesses the donor sequence abundance for each gene in each strain, and tests whether the abundance differs significantly between before and after induced incubation by a negative binomial model (Li et al. 2014; Wang et al. 2019). Because abundances of random donor sequences were consistent across different samples, their read counts were used as controls for read-count normalization in each sample. The positive and negative *β*-scores were calculated for each gene in each strain to estimate whether the gene was positively or negatively selected, which represents that disrupting the gene confers a growth advantage or disadvantage, respectively. There were 19.8–84.7% genes negatively selected but only 0–0.02% genes positively selected in ten strains under false dicovery rate (FDR) < 0.05, echoing disruptions of genes generally tend to be deleterious to various extents (fig. 1*F*, supplementary file S4, Supplementary Material online). Hence, the negative *β*-score was used as a proxy for deleterious effects of gene disruption, which was consistent with previous studies (Zhu et al. 2016; Haapaniemi et al. 2018). The value of negative *β*-score ranged from 0 to 1, and a smaller negative *β*-score means a larger fitness effect led by the disruption of the gene (fig. 1*F*).

The negative *β*-scores of the same gene in different strains were comparable. Spearman’s correlation coefficients of negative *β*-scores between different strains ranged from 0.4 to 0.72, which were higher than the values derived from fitness data of gene deletion lines in four genetic backgrounds assessed by clone image (fig. 1*G*, supplementary file S1: fig. S4, Supplementary Material online, Spearman’s correlation coefficients: 0.04–0.39) (Galardini et al. 2019).

### Evaluation of Intraspecies Specificity of Fitness Effect of Disruptions of the Same Genes

**Fig. 2.**
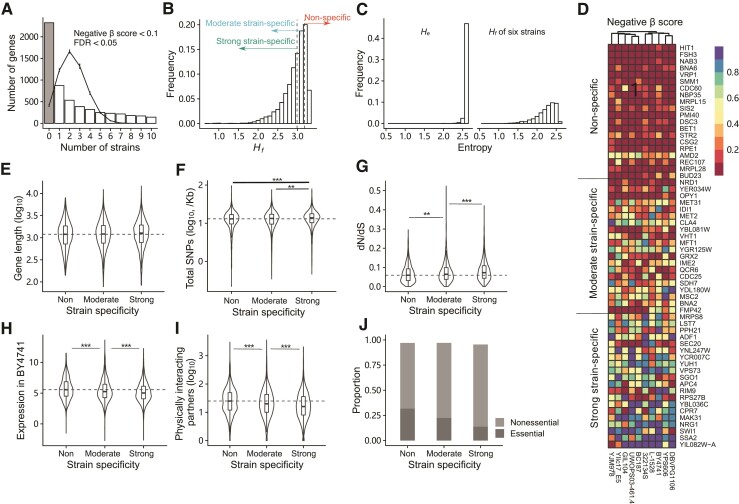
Shannon entropy (*H_f_*) indicates the strain specificity of fitness effect for each gene across different strains. (*A*) Distribution of the numbers of strains in which the disruption of the same gene had similar striking effects (negative *β*-score < 0.1, FDR < 0.05). The line chart with error bars shows the result derived from ten random samplings. (*B*) Distribution of *H_f_* for 5,630 genes. The dashed lines represent *H_f_* = 2.989359 and *H_f_* = 3.177758, which are the thresholds for classifying genes. Genes with *H_f_* smaller than the left threshold are strong strain-specific, and genes with *H_f_* larger than the right threshold are nonspecific. (*C*) Distributions of *H_e_* (left) and *H_f_* derived from six strains (right) of 3,661 overlapped genes. *H_f_* derived from six strains was calculated by negative *β*-scores in six strains randomly sampled from ten strains. The samplings were performed ten times, and the average value of *H_f_* was taken as *H_f_* of six strains for each gene. (*D*) Heatmap for negative *β*-scores of 60 genes with different strain specificity. For each level of strain specificity, 20 genes were sampled randomly. (*E*) Comparison of sequence length of genes with different strain specificity of fitness effects. The information of gene length was obtained according to S288C annotation. The statistic differences of sequence properties between each two groups was examined by Wilcoxon rank-sum test (**: *P* < 0.01; ***: *P* < 0.001). The dashed lines represent the median values for nonspecific genes. (*F*) Comparison of total SNPs per Kb for genes with different strain specificity of fitness effects. (*G*) Comparison of dN/dS of genes with different strain specificity of fitness effects. (*H*) Comparison of expression level of genes with different strain specificity of fitness effects. The expression profile was obtained in BY4741. (*2I*) Comparison of number of physically interacting partners of genes with different strain specificity of fitness effects. (*J*) Proportions of essential and nonessential genes in genes with different strain specificity of fitness effects.

More than 58% of genes (3305/5630) significantly affected cell fitness in at least one strain (negative *β*-score < 0.1, FDR < 0.05, fig. 2*A*). The numbers of strains in which the disruption of the same gene had similar significant effects were counted, and the distribution was very different from random samplings (see Materials and Methods, fig. 2*A*). Only ∼15% of them (494/3305) had similar effects in over eight strains, whereas 26% (872/3305) showed exclusive effects in one strain. To evaluate the strain specificity of the fitness effects for genes, the Shannon entropy was introduced in light of its success in analyzing tissue-specific genes (Schug et al. 2005). The Shannon entropy (*H_f_*) was calculated for each gene by using the negative *β*-scores in all strains (see Materials and Methods, fig. 2*B*). Smaller *H_f_* values mean stronger strain specificity, that is, larger variability within species. Next, we calculated the Shannon entropy of gene expression (*H_e_*) from the expression profiles of six *S. cerevisiae* strains measured in Yang et al.’s (2017) study (see Materials and Methods, supplementary file S5, Supplementary Material online). To remove the influence of number of strains, we also calculated the *H_f_* of six strains by random samplings. Intriguingly, *H_e_* showed a much narrower distribution than *H_f_* (fig. 2*C*). It indicates that genes have more comparable strain specificity of expression than that of fitness effect. The intraspecies variability seemed to be increased from gene expression to the fitness effect of gene disruption.

Taking advantage of comprehensive studies on *S. cerevisiae* strains (especially S288C), we examined several properties of the sequence, evolution, and functions of these genes. Despite low correlation coefficients (absolute value of Spearman’s correlation coefficient < 0.15), certain correlations between *H_f_* and these properties indeed existed (*P* << 0.01). To make it more informative, genes were classified into nonspecific, moderate strain-specific, and strong strain-specific groups by two thresholds of *H_f_* derived by simulations (fig. 2*B*, see Materials and Methods). We excluded 204 genes whose disruptions were barely negatively selected in all strains (negative *β*-score > 0.6 in all strains). The Gene Ontology (GO) analysis of these genes did not reveal any significant terms, and nearly a quarter of them (50/204) have no annotations (supplementary file S4, Supplementary Material online). Genes in the nonspecific group (1200/5630, 21.3%) induced ubiquitous and similar fitness effects in all strains, genes in the strong strain-specific group (2384/5630, 42.3%) had large fitness effects in a small number of strains, and genes in the moderate strain-specific group (1842/5630, 32.7%) were in between (fig. 2*D*).

There were no differences in gene length (subject to S288C) between genes in nonspecific and strain-specific groups (fig. 2*E*). The total number of SNPs relative to S288C was counted for each gene. The total numbers of SNPs for strong strain-specific genes were more than the values for nonspecific and moderate strain-specific genes (fig. 2*F*). It indicated that strong strain-specific genes had the largest variability of sequence. Thus, it is no surprise that strong strain-specific genes suffered the weakest selective pressures when examining gene evolutionary rates derived from four *Saccharomyces* species (Wall et al. 2005) (fig. 2*G*). Using expression profiles in BY4741, YPS606, and BC187 (Yang et al. 2017), we found strong strain-specific genes generally had the lowest expression levels (fig. 2*H*, supplementary file S1: fig. S5, Supplementary Material online). In addition, strong strain-specific genes tended to have fewest physically interacting partners (fig. 2*I*). According to the current essential genes annotated in *S. cerevisiae* strains, the proportion of essential genes decreased from nonspecific, moderate strain-specific to strong strain-specific genes (fig. 2*J*). These results indicate that the strain specificity of the fitness effect for a gene is connected with its evolutionary and functional importance.

### Relationship Between the Strain Specificity of Fitness Effect and Gene Age

**Fig. 3.**
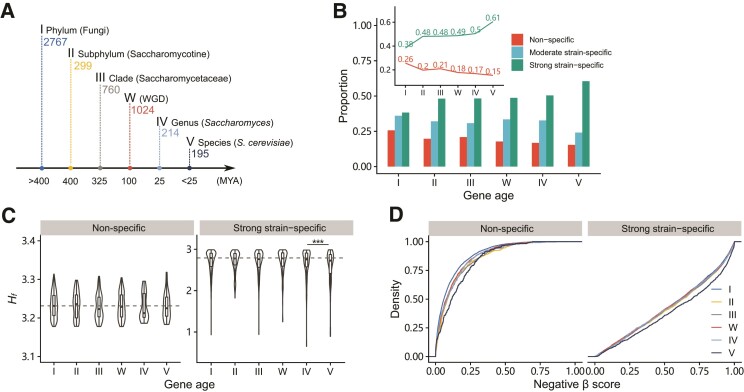
Younger genes tend to have stronger strain specificity of fitness effect. (*A*) A simplified timeline for classifying genes into different age groups, according to Doughty et al.’s (2020) study. The number of genes of each age is labeled. (*B*) Proportions of genes with different strain specificity of six age groups. In particular, the values of proportions for nonspecific genes and strong strain-specific genes were shown in the inset. (*C*) *H_f_* of nonspecific genes (left) and strong strain-specific genes (right) of different age groups. There is no difference among nonspecific genes of different ages. Strong strain-specific genes of age V have significantly lower *H_f_* than those of age I (Wilcoxon rank-sum test, *P* = 0.006). (*D*) Cumulative distributions of negative *β*-scores for nonspecific genes (left) and strong strain-specific genes (right) of different age groups. The cumulative distributions were drawn by negative *β*-scores in ten strains. For nonspecific genes, there are no differences among genes of ages II, III, IV, and V groups, but significant differences exist between genes of age I and age II groups, and between genes of age IV and age V groups (Wilcoxon rank-sum test, I vs. II: *P* = 6.7 × 10^−9^; IV vs. V: *P* = 0.01). For strong strain-specific genes, there are no differences among genes of ages I, II, III, IV, and V groups, but significant differences exist between genes of age IV and age V groups (Wilcoxon rank-sum test, IV vs. V: *P* = 2.7 × 10^−4^).

New genes are always considered to contribute species-specific phenotypic traits (Kaessmann 2010). To explore the role of gene age in the strain specificity of fitness effects, genes were assigned to different ages, according to Doughty et al.’s (2020) study (fig. 3*A*). Genes of age I are ancient genes that are conserved in filamentous fungi, and genes of age V are newly acquired in *S. cerevisiae* species (i.e., *S. cerevisiae* species-specific genes). In the progression from ancient genes to young genes, the proportions of strong strain-specific genes increased, and the proportions of nonspecific genes decreased (fig. 3*B*). This suggests that gene age is genuinely relevant to the strain specificity of the fitness effect. A remarkable observation was that there was no difference in *H_f_* for nonspecific genes of different ages (fig. 3*C* left), though strong strain-specific genes of age V had significantly lower *H_f_* than those of age I (fig. 3*C* right). This provides evidence that a portion of new genes, that is, the nonspecific genes of age V, can rapidly evolve to perform universal functions within species.

We further examined the fitness effect size of genes of each age. As expected, for nonspecific genes, significant differences in negative *β*-scores existed between genes of age I and age II, and between genes of age IV and age V (fig. 3*D* left). That is, for these genes, the size of the fitness effect generally decreased from ancient genes to young genes, echoing the different functional importance of genes of different ages. However, for strong strain-specific genes, there were no differences in negative *β*-scores among genes of ages I, II, III, W, and IV, whereas significant differences existed between genes of age IV and age V (fig. 3*D* right). In other words, the size of the fitness effect cannot be distinguished among genes of all ages, except *S. cerevisiae* species-specific genes. It seemed that strong strain-specific genes might have a ceiling of fitness effect size, which is not affected by gene age, and *S. cerevisiae* species-specific genes are still evolving and have not yet reached this ceiling. To avoid bias from small fitness effects, we excluded the genes with negative *β*-scores larger than 0.3 in ten strains, and we continued to observe similar patterns (supplementary file S1: fig. S6, Supplementary Material online).

### Diverse Strain Specificity of the Fitness Effects for Genes Whose Products Form Complexes

**Fig. 4.**
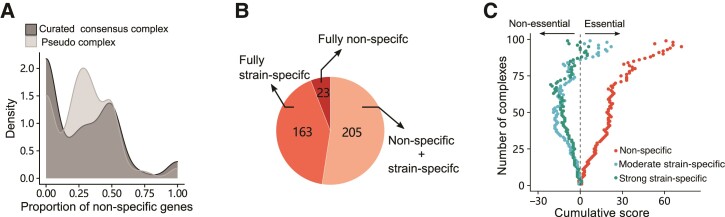
Diverse conservation of members in complexes. (*A*) Density of proportion of nonspecific members for 391 complexes. The curated consensus complex set is indicated by dark gray, and the pseudocomplex set is colored by light gray. (*B*) Three-hundred-ninety-one complexes are classified into three categories, which are fully nonspecific members, fully strain-specific members, and nonspecific/strain-specific members mixed, respectively. (*C*) Cumulative scores for the essentiality of nonspecific, semistrain-specific, and strong strain-specific members in partially essential complexes consisted of both nonspecific and strain-specific members, respectively. The score of an essential gene was defined as 1, and the score of a nonessential gene was defined as −1. For each complex, the essentiality of each member was judged, and scores were calculated for each level of strain specificity, respectively. The cumulative scores were calculated by traversing all complexes consisting of genes with three levels of strain specificity.

Protein complexes are vital for many conserved biological processes (Wan et al. 2015). However, studies have found that members constituted a complex may not be equal in conservation and essentiality (Ryan et al. 2013; Caufield et al. 2015). We wondered whether genes whose products form a protein complex would have identical strain specificity of fitness effect. The levels of strain specificity for members of 391 curated consensus complexes (involving 1,556 genes) were examined (see Materials and Methods, supplementary file S6, Supplementary Material online). Unexpectedly, inconsistent performances of members within complexes were prevalent across 391 complexes (supplementary file S1: fig. S7, Supplementary Material online). To assess the diversity with complexes, the same number of pseudocomplexes was generated by random samplings from genes involved in complexes (see Materials and Methods). The proportion of nonspecific genes in each complex was calculated for both complex sets. Distributions of proportions for the two sets were different. In the pseudocomplex set, the highest peak was approximately at 0.28, which matches the proportion of nonspecific genes in complex genes (0.28, 431/1556) (fig. 4*A*). In contrast, in the curated consensus complex set, the highest peak was at zero. That was to say, a prominent feature in this set was that a relatively high number of them (0.42, 163/391) consisted entirely of strain-specific genes (fig. 4*A*). This result indicates that the diverse strain specificity of fitness effects among members might have biological significance, rather than being merely random combinations.

Among 391 complexes, 23 fully consisted of members encoded by nonspecific genes (fig. 4*B*, supplementary file S6, Supplementary Material online). All of these were formed by relatively older genes (evolved before the divergence of *Saccharomyces*), and 74% (17/23) were formed by genes of the same ages. Additionally, members of such complexes tended to have a large number of physically interacting partners (supplementary file S6, Supplementary Material online). For instance, *BRE5* and *UBP3*, whose products belong to the Ubp3–Bre5 complex, have 394 and 454 physically interacting partners, respectively. In contrast, 163 complexes fully consisted of strain-specific members (including moderate strain-specific and/or strong strain-specific members) (fig. 4*B*, supplementary file 6, Supplementary Material online). Not surprisingly, over 71% of these complexes (116/163) consisted of only nonessential genes annotated in *S. cerevisiae*, and ∼50% (81/163) of them consisted of genes in at least two age groups.

Beyond that, over half of the complexes (205/391) consisted of both nonspecific and strain-specific members (fig. 4*B*, supplementary file S6, Supplementary Material online). Though complexes with only nonessential members were more abundant than those with only essential members in this category (69 vs. 35), in the remaining 101 complexes showing partial essentiality, nonspecific members are apparently enriched in essential genes relative to strain-specific members (fig. 4*C*). The inconsistent performances of members might reflect their different roles when complexes execute functions. For example, in the nucleoporin Nsp1 subcomplex, the FG-nucleoporin components of the nuclear pore complex (*NSP1*, *NUP57*, and *NUP49*) are nonspecific, and the linker nucleoporin component (*NIC96*) is strong strain-specific (Schrader et al. 2008). In the nascent polypeptide-associated complex, the member that showed a strongly strain-specific fitness effect (*BTT1*) is the paralog of one of the moderate strain-specific members (*EGD1*), which indicates genetic redundancy in complexes. Taken together, these results suggest that the constitution of protein complex is far more flexible in terms of fitness effects of disruptions of member genes within species.

## Discussion

This study presents genome-wide fitness effect data for ten *S. cerevisiae* strains, which provide direct and detailed insights into intraspecies variations of mutant phenotypes. Though there was extremely high conservation in DNA sequences for ∼5,600 orthologs, strain-specific variations in fitness effects were detected for most genes. The fraction of nonspecific genes (21.3%) is similar to the fraction of conserved growth profiles in four genetic backgrounds of yeast (9–24%) (Galardini et al. 2019). It is worth noting that growth behaviors in galactose condition differ among these strains. The relative proliferation rates of nine strains compared with BY4741 range from 1.04 to 1.54 with a median of 1.36 (supplementary file S1: fig. S8, Supplementary Material online). Deleterious mutations in more-fit strains have more strongly deleterious effects (Johnson et al. 2019). The different fitness of wildtype strains may be associated with the less conservation of fitness effect.

The degree of strain specificity of a gene is related to its functional and evolutional properties, such as expression level, essentiality, physically interacted partner, and power of natural selection. In particular, we found that strong strain-specific genes have a growing proportion of newly evolved genes. This is consistent with the observations for new genes: they often contribute to lineage- or species-specific phenotypes (Kaessmann 2010; Chen et al. 2013). In fact, nearly half of newly evolved strong strain-specific genes (of age V, 58/118) are unannotated in S288C, indicating that they may be specifically functional in other strains. In addition, because they have been sustained in the long evolution process, ancient genes are usually considered the most important, and their deletion would lead to a large fitness effect (Chen et al. 2012; Yin et al. 2016). This is true for nonspecific genes, but not for strong strain-specific genes (fig. 3*D*), providing an intriguing insight into the functional evolution of genes.

Negative *β*-score was used to indicate the deleterious fitness effect of gene disruption in this study. To assess its capacity, we experimentally examined the deletion effects of eight genes in BY4741 by replacing the target genes with a URA3 marker, respectively (see Materials and Methods). Although the deletion of *SAK1* resulted in an unexpected growth defect, the deletion mutants of BY4741 generally grew in line with their negative *β*-scores (supplementary file S1: fig. S9*A*, Supplementary Material online). We also deleted two genes with distinct negative *β*-scores in the other nine strains (supplementary file S1: fig. S9*B*, Supplementary Material online). Negative *β*-scores of *PHO3* were larger than 0.8 in all strains except Yllc17_E5, and the *PHO3* deletion mutants had near-normal growth in most strains. Conversely, *CKB2* exhibited small negative *β*-scores in all strains, and its deletion had large effects on growth. We further examined the correlation between negative *β*-scores and public fitness data of gene deletion lines in genetic background of S288C assessed by Bar-seq and clone image, respectively (Qian et al. 2012; Galardini et al. 2019). The negative *β*-score as a proxy for fitness effect of gene disruption could not directly indicate the fitness effect of gene deletion (Spearman’s correlation coefficients *ρ* = 0.11 for Bar-seq, *ρ* = 0.05 for clone image), although genes whose deletions resulted in low fitness tended to have less negative *β*-scores (supplementary file S1: fig. S9*C*, Supplementary Material online). This may due to the intrinsic difference between gene deletion and gene disruption. Different culture conditions (carbon source, culture medium) and different measurements of fitness in different studies may also have contributions.

However, neither a clustering tree derived from fitness effects of all genes nor clustering trees of genes in each level of strain specificity exactly agreed with the phylogenetic tree of these strains (supplementary file S1: fig. S10, Supplementary Material online). The discordance between genomic divergence and phenotypic variation within species is not rare, and sporadic cases have been observed in plants, vertebrates, reptiles, and fungi (Yvert et al. 2013; Jin et al. 2014; Valladares et al. 2014; Harris et al. 2018). Recent views state that phenotypic evolution may obey different laws of genotypic evolution (Zhang 2018; Wideman et al. 2019). We indeed observed that the variability of fitness effects within species was larger than that of gene expression (fig. 2*C*), which probably is an intrinsic feature for mutant phenotype. Vu et al. (2015) suggested that natural variation in gene expression can significantly affect the severity of mutant phenotypes, and loss of function of genes with a 20% difference in expression level compared with the species average would probably lead to severe phenotypes.

Genome-wide CRISPR–Cas9 screening is a general tool for studying the essentiality of genes and other functional elements (Fei et al. 2019; Ihry et al. 2019; Schwartz et al. 2019; Choudhury et al. 2020). Nevertheless, it is difficult to give a definite cutoff to distinguish essential and nonessential genes on a genome-wide scale no matter which indicator is used (Shalem et al. 2014; Morgens et al. 2016; Wang et al. 2017; Ihry et al. 2019). An intrinsic reason for this is that some genes are only essential in certain conditions (Papp et al. 2004; Liu et al. 2015; Chen et al. 2016; Larrimore and Rancati 2019), and mutants of some nonessential genes can severely influence cell growth (e.g., some ribosomal genes). Another important reason is the library-specific false negatives brought by inactive gRNAs (Ong et al. 2017; Schwartz et al. 2019). Here, pleiotropic mutational effects, which are either ad hoc or evolutionarily selected, may also contribute to phenotypic variations (He 2016; Liu et al. 2020). Under these circumstances, the Shannon entropy provides a reliable estimation of the effective size and conservation of fitness effects for each gene by considering their whole performances in ten strains, which can partly reduce the impact of false negatives in a single strain (He 2016).

Here we systematically investigated the intraspecies variation in fitness effects of 5,630 gene mutants in ten *S. cerevisiae* strains using CRISPR–Cas9 screening. We showed that the variability of fitness effects induced by gene disruptions is very large within species, which supports that phenotypic evolution may obey different laws of genotypic evolution. Importantly, the strain specificity of fitness effect of a gene is connected to its functions and evolutionary time. This work is a comprehensive evaluation of intraspecies phenotypic variation, which provides crucial insights for gene function prediction and biological complexity.

## Materials and Methods

### Yeast Strains and the Composition of Vector

Twenty-five *S. cerevisiae* strains were included in this study. Except for BY4741 (*MATa*, *his3*, *leu2*, *met15*, *ura3*) and GIL104 (a haploid yeast strain derived from the W303 background, *MATa*, *URA3*, *leu2*, *trp1*, *CAN1*, *ade2*, *his3*, *bar1Δ::ADE2*) (Lang and Murray 2008), other strains (*MATa*, *ura3*) were purchased from the National Collection of Yeast Cultures. The wildtype *URA3* in GIL104 was first replaced by a *LEU2* cassette. All strains were cultured at 30 °C.

The background vector used in this study is pYES2 (Invitrogen). A gRNA expression cassette and a Cas9 expression cassette were constructed into the vector. The former consisted of the *SNR52* promoter, sgRNA, gRNA scaffold, *SUP4* terminator, and a 100-bp donor sequence with 8-bp deletion around the PAM sequence. The latter included the *GAL1* promoter, two SV40 nuclear localization signal sequences, Cas9, and the *CYC1* terminator. Codon optimization was performed for Cas9 (derived from Streptococcus pyogenes) by DNA Chisel (Zulkower and Rosser 2020), and the optimized Cas9 was synthesized by Genewiz and cloned into pYES2.

### Verify the Efficiency of the CRISPR–Cas9 System

To verify the typical gene disruption efficiency of this system in 25 *S. cerevisiae* strains, a 20-bp guide sequence targeting 157 bp downstream of the start codon of *ADE2* with the corresponding donor sequence was constructed into the vector for *ADE2* disruption (Bao et al. 2015). The constructed vector was transformed into each strain according to the standard polyethylene glycol (PEG)/LiAc-based method, as described in our previous study (Liu et al. 2020). Transformants were growth in plates of synthetic medium deprived of uracil (SC-URA, 2% glucose, 2% agar) for 2 days. Then the positive clones were confirmed by polymerase chain reaction (PCR), and cultured in synthetic medium deprived of uracil (SC-URA, 2% glucose) for 2 days with shaking. After that, ∼10^4^ cells were transferred to synthetic medium deprived of uracil containing galactose (1% galactose, 2% raffinose) and incubated for 2–3 days with shaking. To examine the editing events, dilutions of 10 μl cells were spread on YPD plates (1% yeast extract, 1% peptone, 2% dextrose, 2% agar) for 2–3 days, and fractions of red clones were counted for each strain.

### CRISPR–Cas9 Library Design

To design the CRISPR library, the potential sgRNAs were predicted for each gene of S288C by sgRNAScorer.2.0 (Chari et al. 2017), and the efficacy of each sgRNA was evaluated by CRISPRseek (Zhu et al. 2014). SgRNAs with top5OfftargetTotalScore larger than 15 and gRNAefficacy <0.3 were excluded (top5OfftargetTotalScore and gRNAefficacy: two indices derived from CRISPRseek). SgRNAs, including SNPs of *S. cerevisiae* strains, were also excluded (Bergström et al. 2014). Two criteria were used to pick five sgRNAs per gene: the position of the sgRNAs should be in the first half of the ORFs, and the gRNAefficacy should be as high as possible. The first criterion was relaxed when insufficient sgRNAs could be obtained, and extra sgRNAs were supplemented to increase the editing opportunities of these genes. For each sgRNA, 50-bp homologous sequences on each side of its PAM sequence with a centered 8-bp deletion were detected and connected up as its donor sequence. Then, we got 28,457 sgRNAs with donor sequences for 5,706 genes. We also designed 100 random sgRNAs, each with 100-bp random donor sequences for control.

### Library Production

The oligonucleotide library of sgRNAs with *SUP4* terminator and donor sequences was synthesized by Genewiz. BbSI and EcoRI restriction sites were appended to each sequence. First, the library was amplified by PCR (F primer: TGACGCGAAGACATGATC; R primer: GCGAATTCCACTCAGTCC) and cloned into PUC19 containing *SNP52* promoter using restriction enzymes BbSI (NEB #R3539), EcoRI (NEB #R3101), and T4 ligase (NEB #M0202). Next, the gRNA scaffold was cloned into the above vector by seamless DNA cloning (Clonesmarter, Seamless Assembly Cloning Kit # C5891-50). Briefly, the vector in the first step was linearized by PCR (F primer: GCACACCTGCTTATGTCT; R primer: TAGCTCTAAAACNNNN); the gRNA-scaffold was amplified using primers with overlapped sequences (F primer: NNNGTTTTAGAGCTAGAAATAGCAAGTTAAAATAAGGCTAGTCCGTTATCAACTTGAAAAAGTGGCACCGAGT; R primer: AGACATAAGCAGGTGTGCAGACATAAAAAACAAAAAAAGCACCGACTCGGTGCCACTTTTTCA). The library of gRNA expression cassette was obtained by the above two steps. Third, both pYES2 with the Cas9 expression cassette and the library vector with gRNA expression cassette were digested with SphI (NEB #R3182) and EcoRI (NEB #R3101). Then, the gRNA expression cassette fragments were constructed into pYES2 with the Cas9 expression cassette using T4 ligase. In each round of cloning, the ligation mixture was purified by ethanol precipitation and transformed into DH5α electrocompetent *Escherichia coli* cells (Weidibio, CAT#DE1001) by electroporation. The transformants were verified using PCR, and the positive rates were achieved to 100% in all rounds. About 10^6^ transformants were collected, and plasmids were extracted in each round. To confirm the coverage of sgRNAs in the plasmid library, the donor sequences were amplified by PCR (F primer: GTCTGCACACCTGCTTATGTC; R primer: GAGGTCGCTCTTATTGACCAC). High-fidelity DNA polymerase (TOYOBO, KOD-FX 101) was used, and multiple amplifications were performed parallelly to reduce batch effects. Then, the amplicons were sequenced on a HiSeq platform at Genewiz with two technical replicates. NGS of the library captured 97.3% of the designed donor sequences (27,799/28,557), which covered ∼100% of the targeted genes (5705/5706).

### CRISPR–Cas9 Screening in Various Strains

The plasmid library was transformed into each yeast strain by a high-efficiency PEG/LiAc-based method (Takara, Yeastmaker™ Yeast Transformation System 2). SC-URA plates were used to select the cells successfully taking up plasmids. The transformants were verified using PCR, and the positive rates were achieved to 100%. To assess the probability of a transformant with more than one vector, we picked 40 single clones from four strains (BY4741, 322134S, DBVPG1106, L-1528), and amplified and sequenced the sgRNA sequences. If the sgRNA sequence of a clone was not unique, it was defined as a hybrid event. Sequencing of ∼40 transformants revealed that the probability of a transformant with more than one vector was <0.08. Over 5 × 10^7^ transformants were collected for each strain. To determine the baseline of the abundance of donor sequence for each strain, about 10^9^ cells were immediately taken from the collections, and donor sequences were amplified following sequencing, as described above. Meanwhile, ∼5 × 10^7^ cells were taken from the collections and incubated in a liquid synthetic medium deprived of uracil containing galactose to induce the expression of Cas9 for 2–3 days with shaking. To ensure each sgRNA has the opportunity to work, the induced incubation was performed in ten 96-well plates for each strain. Each well was an independent environment. After induced incubation, all cells were collected, mixed well, and transferred into a fresh liquid synthetic medium deprived of uracil with glucose for 1–2 days with shaking. Then, the cells were collected and used to extract plasmids. Donor sequences were amplified and sequenced, as described above. Each condition in each strain had two replicates. The sequencing data were in BioProject (PRJNA693154). For each strain, there were four samples, including two samples before edited (replicate_1_control and replicate_2_control) and two samples after edited (replicate_1_treatment and replicate_2_treatment).

### Data Processing and Analysis

Reads were mapped to the custom index built according to relationships between donor sequences and genes using BWA (Version 0.7.17) (Li and Durbin 2009). The coverages of donor sequences ranged from 90.6% to 95.2% for plasmids collected before induced incubation in various strains. MAGeCK (Version 0.5.9) (Wang et al. 2019) was used to quantify the variations of abundances of donor sequences with the following key parameters: remove-zero-threshold 30, norm-method control − control-sgrna random_donor_sequences.txt. The txt file recorded the names of random donor sequences. To remove the effects of different total reads in different sample, the counts of random donor sequences were used as controls for normalization of CRISPR screens because their variations reflected the background effect of the vector. There were 92.3–99.6% genes having fold-change values <1 in different strains, suggesting the successful generation of mutants by this system and the occurrence of negative selection. The value of negative *β*-score (representing the strength of negative selection) was calculated by ranking sgRNAs on the basis of their *P*-values calculated from the negative binomial model and using a modified robust ranking aggregation algorithm by MAGeCK. The negative-selection data of genes in each strain are listed in supplementary file S4, Supplementary Material online.

The numbers of genes with significant effects were counted for each strain with the thresholds of FDR < 0.05 and negative *β*-score < 0.1. The numbers of strains in which the disruption of the same gene had similar significant effects were counted (i.e., the number of cooccurrence strains). To evaluate the randomness of the distribution of the numbers of cooccurrence strains, a certain number of genes were sampled for each strain, and the numbers of different strains were different according to the number of genes with significant effects in each strain, respectively. Then the numbers of cooccurrence strains were counted. The random sampling performed ten times, and the average number and standard variation were calculated for each condition.

There were 5,630 genes having negative *β*-scores in all strains. To evaluate the conservation of fitness effect for each gene across different strains, Shannon entropy (*H_f_*) was calculated for each gene as following (Schug et al. 2005). We defined the fitness effect level of gene *i* in strain *j* as *f*_*i*,*j*_ = −log _10_*β*_*i*,*j*_, where *β*_*i*,*j*_ is the negative *β*-score. The mathematical treatment of negative *β*-score could expand the difference between genes, considering a fairly large number of genes had values <0.1 (e.g., 23% in BY4741). Then, the relative fitness effect of gene *i* in strain *j* is $p_{\;j|i}=f_{i,j}/\;\sum_{1\leq j\leq N}\;f_{i,j}$, where *N* is the number of strains (*N* = 10). The entropy of fitness effect distribution (*H_f_*) for gene *i* is $H_{\;fi}=-\;\sum_{1\leq j\leq N}\;p_{\;j|i}\log_{2}\;(\;p_{\;j|i})$.

To define the threshold of different levels of *H_f_*, simulations were performed to model a gene with uniform fitness effects in all strains following the previous study (Schug et al. 2005). The fitness effects of the gene in different strains were assumed to fluctuate around an average fitness effect with deviations following a narrow distribution. Average fitness effects were sampled from the average values of 5,630 genes. To model a gene having a highly conserved fitness effect, we set the deviation to 1 (i.e., fold changes between the fitness effect of a particular strain and the average level were within 1.5 in over 70% strains). The value of *H_f_* was estimated by 2,000 random samplings, and the average value of ten repeats was used to define the threshold. To get a looser threshold, the deviation was set to 1.5.

The expression profiles of six *S. cerevisiae* strains were derived from Yang et al.’s (2017) study, and the entropy of expression of each gene (*H_e_*) was calculated as follows:

$$H_{ei}=-\underset{1\leq i\leq 6}{\sum }\;p_{\;j|i}\log_{2}\;(\;p_{\;j|i})$$

where $p_{\;j|i}=\frac{\log_{2}r_{i,j}}{\sum_{1\leq j\leq 6}\;\log_{2}r_{i,j}}$, and *r*_*i*,*j*_ is the expression level (log 2 base of RPKM, Reads Per Kilobase per Million mapped reads) of gene *i* in strain *j*. Entropy ranges from 0 to log2(*N*). For comparison, we calculated *H_f_* for 3,661 overlapped genes in six strains sampled from ten strains in this study. The samplings were performed ten times, and the average value of *H_f_* was calculated for each gene.

### Yeast Gene Deletions and Fitness Estimation

Eight genes (*PHO3*, *MPO1*, *YDR132C*, *ZRC1*, *REV3*, *SAK1*, *HSE1*, and *CKB2*) with various negative *β*-scores were replaced by a *URA3* cassette based on homologous recombination in BY4741, respectively. Transformations of gene replacements were according to the standard PEG/LiAc-based method. *PHO3* and *SAK1* were deleted in the other nine strains by the same method. The successful deletion clones were identified by PCR. Because flocculation is widespread in most strains, we used clone size to estimate the fitness effects of deletion lines. For each strain, the wildtype and deletion lines were incubated in a synthetic medium with 2% glucose for 2 days. Then, cell concentrations were measured and quantified to ensure the initial cell number for dilution was the same in all lines. Cells were gradiently diluted (1:100, 1:500, 1:1,000, 1:5,000, 1:10,000), and 1 μl cells in each gradient were dropped to synthetic medium plates. The plates were incubated for 30 h and imaged. The relative proliferation rates of nine strains in galactose (2%) and glucose condition were obtained from Warringer et al.’s (2011) study.

### Gene Features Analysis

The phylogenetic tree of ten *S. cerevisiae* strains and an S. *paradoxus* strain was drawn with the distance matrix derived in West et al.’s (2014) study using MEGA-X (Molecular Evolutionary Genetics Analysis) (Kumar et al. 2018). The fitness of ∼5,000 nonessential gene deletion strains in YPD measured by Bar-seq was from Qian et al.’s (2012) study. The fitness of 3,786 gene knockouts in four *S. cerevisiae* strains in SC-HEPES medium measured by clone image was from Galardini et al.’s (2019) study. Median values of normalized sizes were calculated from replicates for mutants and wild-type strain, respectively. Then the fitness for each mutant was the ratio of the median value of mutants vs. the median value of wild-type strain. Gene annotations were downloaded from *Saccharomyces* Genome Database (SGD, http://sgd-archive.yeastgenome.org/curation/literature/gene_association.sgd), and the representative putative genes were filtered by keywords (“putative” and “unknown”). The GO slim mapping file was downloaded from SGD (https://www.yeastgenome.org/go_slim_mapping.tab), and the representative ribosomal genes were filtered from the terms including “ribosomal” or “ribosome”. The ORF sequences of S288C were downloaded from SGD, and the sequences of other strains were downloaded from *Saccharomyces* Genome Resequencing Project (SGRP, http://www.moseslab.csb.utoronto.ca/sgrp/download.html). The ORF length in S288C was used to represent gene length for each gene. The information of SNPs in *S. cerevisiae* was downloaded from SGRP, and the total number of SNPs compared with the reference genome of S288C was counted for each gene. Gene evolutionary rates were from Wall et al.’s (2005) study, which were estimated from 4,120 orthologs of *S. bayanus*, *S. mikatae*, *S. paradoxus*, and *S. cerevisiae*. The annotations of gene age were from Doughty et al.’s (2020) study. The expression levels of *S. cerevisiae* genes were estimated by the average values of six *S. cerevisiae* strains in Yang et al.’s (2017) study (including YPS606, IFO1815, DBVPG6040, Y9, BC187, and YJM145). Physically interacting partners for each gene were extracted from the BioGRID database (https://thebiogrid.org/BIOGRID-ORGANISM-Saccharomyces_cerevisiae_S288c-3.5.169). The list of essential and nonessential genes in S288C (genes with inviable and viable phenotypes, respectively) was downloaded from SGD on November 2, 2020. Information of protein complexes was obtained from Benschop et al.’s (2010) study, and the curated consensus complex list was used. To generate a pseudocomplex set, in each sampling, the number of members was randomly sampled from the numbers in the curated consensus complex set, and the corresponding set of genes were randomly sampled from complex genes. All features of genes are listed in supplementary file S4, Supplementary Material online.

## Supplementary Material

Supplementary data are available at *Genome Biology and Evolution* online.

## Author Contributions

L.L. and X.H. designed the study. Y.W., B.J., Y.W., and L.L. carried out experiments. L.L. and Y.W. analyzed data. L.L. wrote the article with inputs from all authors.

## Supplementary Material

evac061_Supplementary_Data

## Data Availability

The donor sequence abundance data generated in this study are available in the NCBI BioProject database (https://www.ncbi.nlm.nih.gov/bioproject/) under accession number PRJNA693154.
