# Supplementary material for: Rapid Intraspecies Evolution of Fitness Effects of Yeast Genes
Source: Genome Biol Evol. 2022 Apr 28;14(5):evac061. doi: 10.1093/gbe/evac061 (PMC9113246; doi:10.1093/gbe/evac061)
Supplement: evac061_Supplementary_Data [file evac061_supplementary_data.zip › Supplementary file 1_R2.pdf]

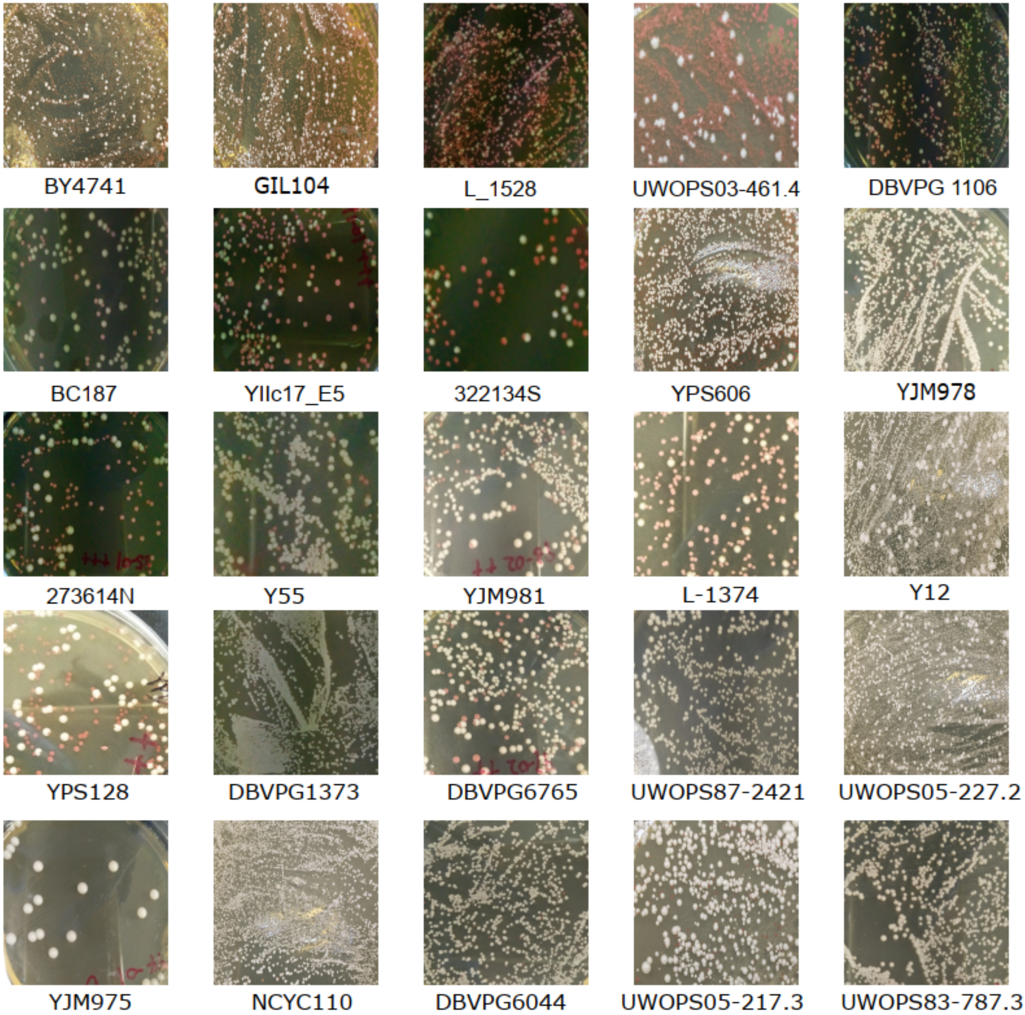
BY4741
GIL104
L_1528
UWOPS03-461.4
DBVPG 1106
BC187
YIIc17_E5
322134S
YPS606
YJM978
273614N
Y55
YJM981
L-1374
Y12
YPS128
DBVPG1373
DBVPG6765
UWOPS87-2421
UWOPS05-227.2
YJM975
NCYC110
DBVPG6044
UWOPS05-217.3
UWOPS83-787.3

Figure S1

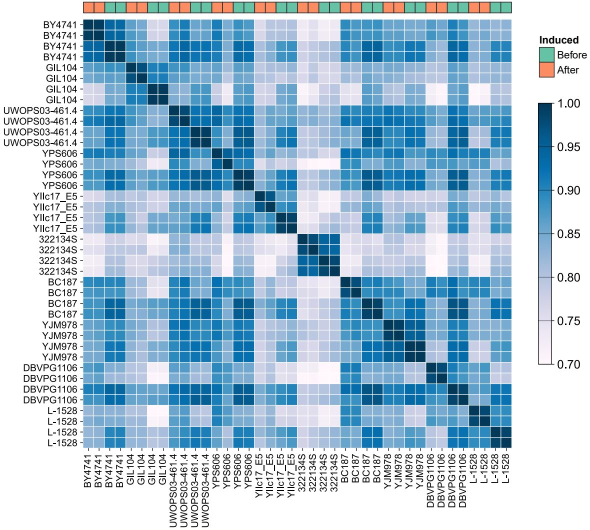
Induced
Before
After
1.00
0.95
0.90
0.85
0.80
0.75
0.70
BY4741
GIL104
UWOPS03-461.4
YPS606
YIlc17_E5
322134S
BC187
YJM978
DBVPG1106
L-1528

Figure S2

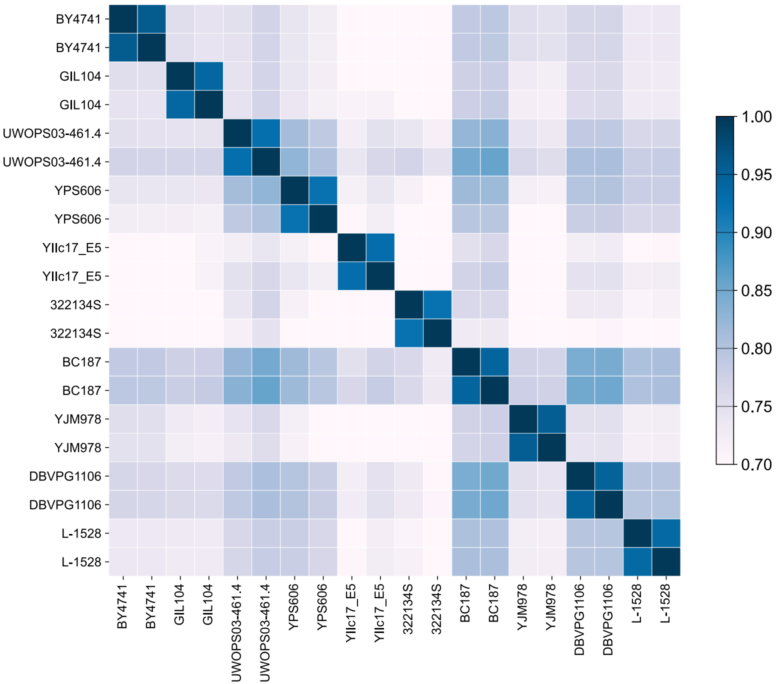

BY4741
BY4741
GIL104
GIL104
UWOPS03-461.4
UWOPS03-461.4
YPS606
YPS606
YIIc17_E5
YIIc17_E5
322134S
322134S
BC187
BC187
YJM978
YJM978
DBVPG1106
DBVPG1106
L-1528
L-1528
1.00
0.95
0.90
0.85
0.80
0.75
0.70

Figure S3

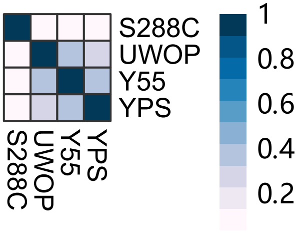

Figure S4

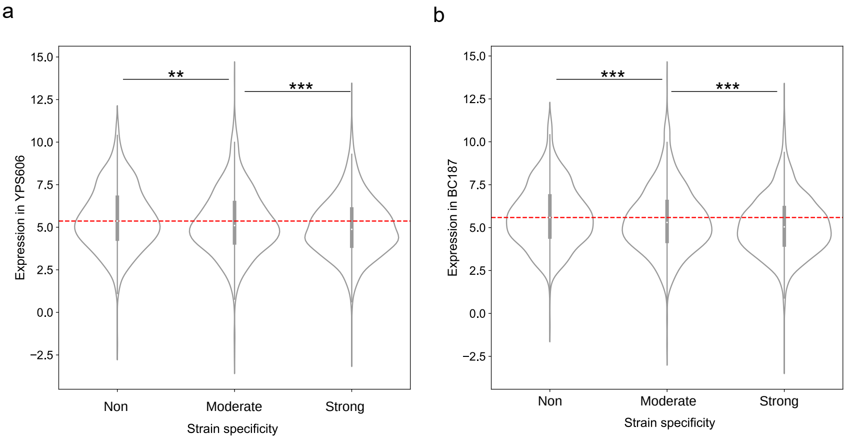
a
b
**
***
***
***
15.0
12.5
10.0
7.5
5.0
2.5
0.0
−2.5
Expression in YPS606
Expression in BC187
Non
Moderate
Strong
Strain specificity

Figure S5

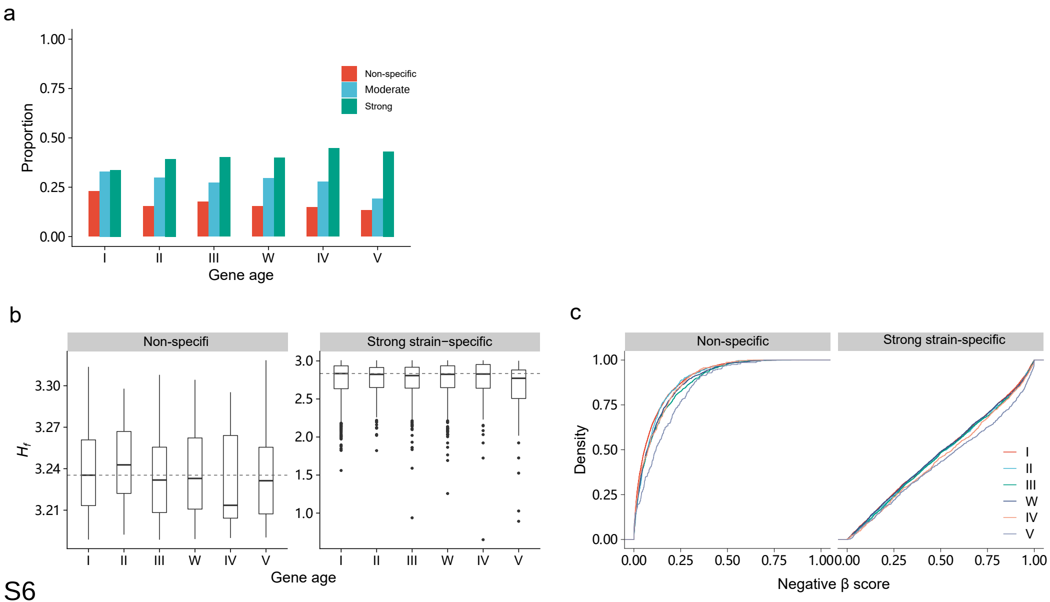

a
1.00
0.75
0.50
0.25
0.00
Proportion
Non-specific
Moderate
Strong
I
II
III
W
IV
V
Gene age
b
Non-specifi
Strong strain−specific
3.30
3.27
3.24
3.21
H_f
3.0
2.5
2.0
1.5
1.0
I
II
III
W
IV
V
Gene age
c
Non-specific
Strong strain-specific
1.00
0.75
0.50
0.25
0.00
Density
0.00
0.25
0.50
0.75
1.00
Negative β score
I
II
III
W
IV
V

Figure S6

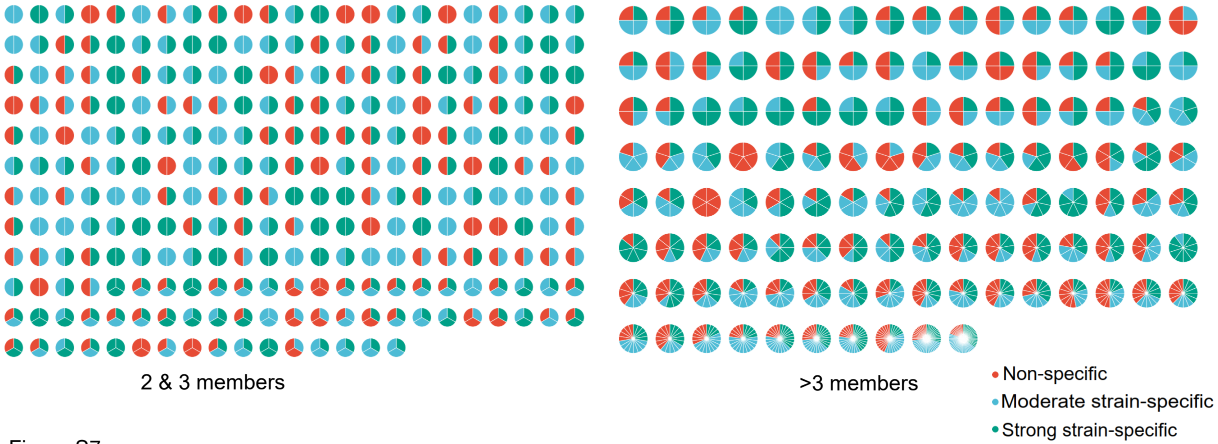
2 & 3 members
>3 members
Non-specific
Moderate strain-specific
Strong strain-specific

Figure S7

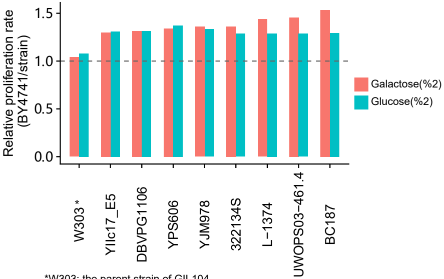

*W303: the parent strain of GIL104

Figure S8

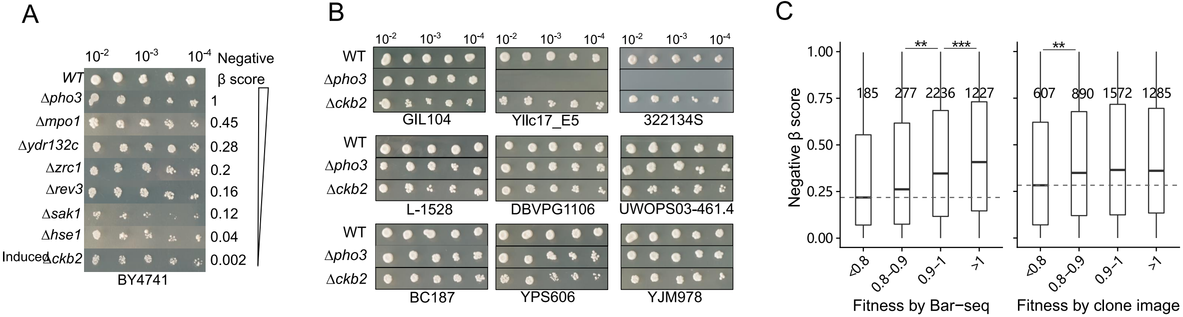
A
10-2
10-3
10-4
Negative β score
WT
Δpho3
1
Δmpo1
0.45
Δydr132c
0.28
Δzrc1
0.2
Δrev3
0.16
Δsak1
0.12
Δhse1
0.04
Induced Δckb2
0.002
BY4741
B
WT
Δpho3
Δckb2
GIL104
Yllc17_E5
322134S
L-1528
DBVPG1106
UWOPS03-461.4
BC187
YPS606
YJM978
C
Negative β score
1.00
0.75
0.50
0.25
0.00
**
***
185
277
2236
1227
607
890
1572
1285
<0.8
0.8–0.9
0.9–1
>1
Fitness by Bar−seq
Fitness by clone image

Figure S9

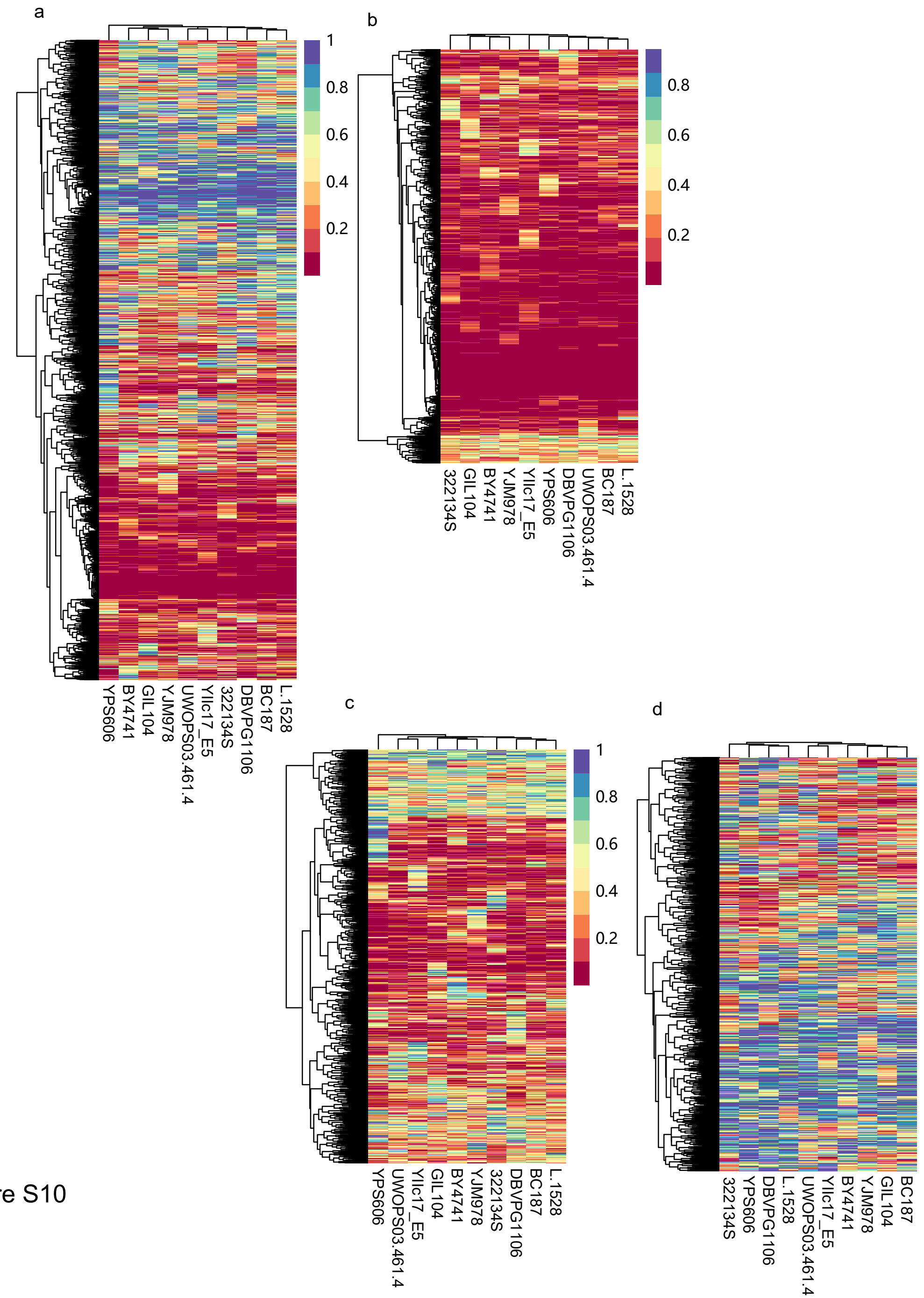
a
1
0.8
0.6
0.4
0.2
YPS606
BY4741
GIL104
YJM978
UWOPS03.461.4
YIIc17_E5
322134S
DBVPG1106
BC187
L.1528
b
0.8
0.6
0.4
0.2
322134S
GIL104
BY4741
YJM978
YIIc17_E5
YPS606
DBVPG1106
UWOPS03.461.4
BC187
L.1528
c
1
0.8
0.6
0.4
0.2
YPS606
UWOPS03.461.4
YIIc17_E5
GIL104
BY4741
YJM978
322134S
DBVPG1106
BC187
L.1528
d
322134S
YPS606
DBVPG1106
L.1528
UWOPS03.461.4
YIIc17_E5
BY4741
YJM978
GIL104
BC187

Figure S10
